# Spatial and Temporal Detection of Ions Ejected from Coulomb Crystals

**DOI:** 10.1021/acs.jpca.3c08132

**Published:** 2024-04-08

**Authors:** Jake A. Diprose, Vincent Richardson, Paul Regan, Adam Roberts, Sergey Burdin, Andriana Tsikritea, Konstantinos Mavrokoridis, Brianna R. Heazlewood

**Affiliations:** † Department of Physics, University of Liverpool, Liverpool L69 7ZE, U.K.; ‡ Department of Physics, TU Dortmund, Dortmund 44227, Germany

## Abstract

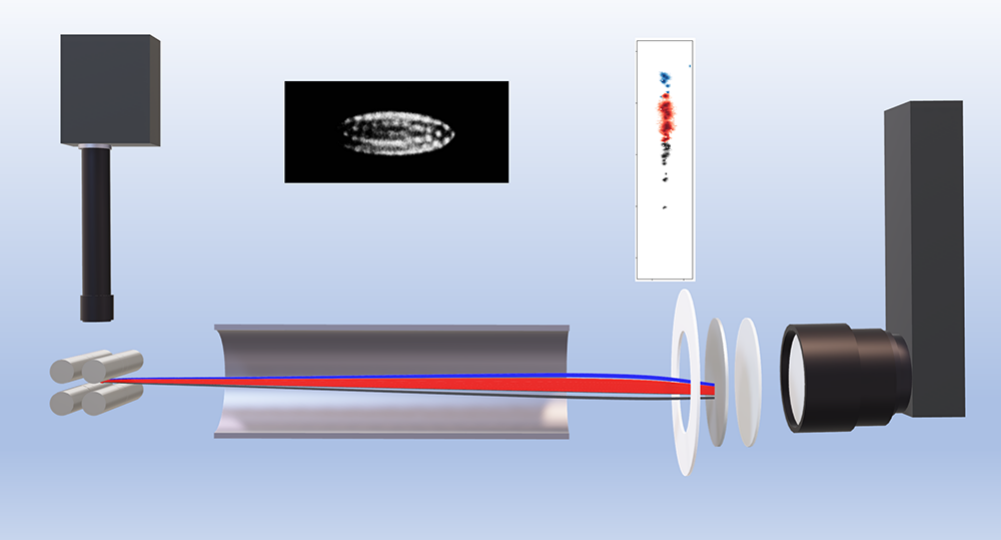

Coulomb crystals have proven to be powerful and versatile tools for the study of ion–molecule reactions under cold and controlled conditions. Reactions in Coulomb crystals are typically monitored through a combination of *in situ* fluorescence imaging of the laser-cooled ions and destructive time-of-flight mass spectrometry measurements of the ejected ions. However, neither of these techniques is able to provide direct structural information on the positions of nonfluorescing “dark” ions within the crystal. In this work, structural information is obtained using a phosphor screen and a microchannel plate detector in conjunction with a Timepix3 camera. The Timepix3 camera simultaneously records the spatial and temporal distribution of all ions that strike the phosphor screen detector following crystal ejection at a selected reaction time. A direct comparison can be made between the observed Timepix3 ion distributions and the distributions established from SIMION simulations of the ion trajectories through the apparatus and onto the detector. Quantitative agreement is found between the measured Timepix3 signal and the properties of Coulomb crystals assigned using fluorescence imaging—independently confirming that the positions and numbers of nonfluorescing ions within Coulomb crystals can be accurately determined using molecular dynamics simulations. It is anticipated that the combination of high-resolution spatial and temporal data will facilitate new measurements of the ion properties within Coulomb crystals.

## Introduction

The use of trapping and laser cooling methods to produce ensembles of translationally cold ions is an area of active research interest. Much of this interest is inspired by the diverse range of applications for cold, confined ions—including their use as qubits,^1,2^ in the determination of fundamental constants,^3,4^ for precision measurements,^5−8^ and for the study of chemical reaction dynamics and kinetics.^9−16^ As many ionic species of interest cannot be easily or efficiently laser-cooled, Coulomb crystals have emerged as a useful tool for the study of processes involving sympathetically cooled trapped ions.

Coulomb crystals can be formed following the laser cooling of trapped ions, where the combination of Coulombic repulsion between neighboring ions and the confining trapping fields leads to the formation of a periodic lattice-like “crystal” structure. The fluorescence emitted by the laser-cooled ions enables the average positions of these species within the crystal to be imaged using devices such as charge-coupled-device (CCD) cameras. Cotrapped ions with appropriate mass-to-charge ratios can be sympathetically cooled by elastic collisions with the laser-cooled ions, yielding multicomponent Coulomb crystals.^17−21^

A number of methods have been developed for the characterization of small Coulomb crystals, with recent examples including the nondestructive quantum state determination of a single cotrapped ion^22^ and the counting of “dark” ions with unitary efficiency (for Coulomb crystals containing no more than eight nonfluorescing ions).^23^ While these approaches offer exceptional precision for the characterization of very small Coulomb crystals, they cannot be straightforwardly applied to the study of larger Coulomb crystals—as typically adopted in reactivity studies.

Instead, reactivity studies typically employ either *in situ* monitoring of crystal evolution through the comparison of fluorescence images with high-level molecular dynamics simulations^10−12^ or the destructive determination of ion numbers from time-of-flight mass spectrometry (ToF-MS) measurements.^24−26^ These methods, used both independently and in combination, have facilitated the successful study of a range of reactions in Coulomb crystals. Fluorescence image comparisons rely on assumptions about the identities of the dark (nonfluorescing) ions, as these species cannot be directly observed by a CCD camera. ToF-MS measurements provide quantitative data on the composition of the crystal at the point of ejection but contain no structural information on where these ions reside within the crystal. Here, we offer a method for detecting the spatial distribution of the ejected ions, enabling Coulomb crystal simulations to be unambiguously validated and revealing important details about the trapping environment and ejection fields.

The coupling of microchannel plates (MCPs) with a phosphor screen allows for the simultaneous recording of ion flight times and positions with the channel-specific electron cascade generated by the MCPs, inducing phosphorescence on the screen. When interfaced with a CCD camera, ion positions can be recorded as a function of time—with this approach widely used in velocity map imaging studies.^27−29^ Variations of the technique have been employed in the detection of Li^+^ ion clouds to obtain radial ion velocity profiles,^30^ in addition to the study of buffer-gas cooling effects on non-neutral plasmas (containing 10^7^ ions) in linear Paul traps.^31^

Advances in camera technology have recently enabled the simultaneous measurement of both the position and timing of ion signals, achieving exceptional time resolution while maintaining position sensitivity. The Timepix3 camera has a hybrid pixel readout chip capable of performing simultaneous time-of-arrival (ToA) and time-over-threshold (ToT) measurements with nanoscale temporal resolution.^32^ Successful applications of the Timepix3 camera span a diverse range of fields, from the detection of cosmic rays with liquid argon time projection chambers to fast mass microscopy and coincidence velocity map imaging measurements.^33−39^

In this work, we employ a Timepix3 camera to monitor a phosphor screen coupled to an MCP stack—facilitating the spatial and temporal detection of ions ejected from Coulomb crystals. By combining the phosphor screen and Timepix3 signals (from the ejected ions) with fluorescence images (from the trapped crystals), we can compare the structural and chemical information obtained from each source. The experimental measurements are combined with trajectory simulations performed using SIMION,^40^ providing a direct link between the experimentally observed fluorescence and ejection data. From this analysis, we demonstrate the suitability of our approach for obtaining quantitative information about the composition and spatial distribution of Coulomb crystals. We explore the impact of the trapping and ejection fields, identifying and rationalizing a positional mass-dependent spatial separation of the ejected ions on the detector.

In order to perform chemical kinetics and dynamics measurements using ToF-MS, a high detection efficiency is required for all of the ionic species of interest. The detection efficiency depends on the identities and numbers of ions in the crystal^24,41^ and is also influenced by the trapping parameters and the extraction voltages adopted. Without spatial information at the detector, it can be incredibly challenging to validate SIMION predictions in some reaction systems—hindering the quantification of ion detection efficiency. As such, previous studies conducted using the apparatus described in this work required thousands of Coulomb crystal molecular dynamics (CCMD) simulations, alongside the recording of ToF-MS measurements under different sets of conditions and thorough investigation of energetic pathways, to ensure that the analysis was robust and that no species were un- (or under-) detected.^11,12^ With the phosphor screen and Timepix3 camera in place, as introduced in this work, it is straightforward to establish where the ions impinge on the detector and to directly compare these distributions with simulations. A quantitative correlation is observed between the signal captured on the phosphor screen and the predicted signal from simulations for Ca^+^ crystals containing up to 600 ions.

The additional positional and timing information provided by the Timepix3 camera—beyond what is recorded in traditional ToF-MS approaches—is therefore important for reaction studies, as it can reveal circumstances when ions in the crystal do not reach the detector. Furthermore, when studying larger crystals (defined in this work as crystals containing >600 ions), it is possible to quantify and optimize the detection efficiency—and to include these details in the subsequent data analysis.

Finally, it is anticipated that the combination of spatial and temporal detection capabilities could facilitate a series of new measurements. Potential applications include establishing the rate of sympathetic cooling for different ionic species and identifying the time scale of ion “migration” through a given crystal (*i.e.*, the movement of ions from where they are initially formed in the trap to their equilibrium position in the crystal). Understanding the efficiency of sympathetic cooling is critical in fields such as quantum information processing, where cooling the computational ions has been described as the “runtime bottleneck”.^42^ While sympathetic cooling has been experimentally measured in very small crystals (with <5 ions),^1,42,43^ it is challenging to apply these techniques to more complex systems, and sympathetic cooling in larger crystals has been predominantly studied using detailed simulations.^44−47^ The time-dependent kinetic energy distribution of laser-irradiated ^113^Cd^+^ ions was recently experimentally measured in large bicomponent Cd^+^–Ca^+^ Coulomb crystals.^48^ However, as this approach was based on the Doppler broadening of the ^113^Cd^+^ fluorescence signal, it cannot be straightforwardly applied to the majority of (nonfluorescing) cotrapped species that are of interest in ion reactivity studies.

Similarly, for crystals containing >10 ions, it is challenging to experimentally probe the positions of nonfluorescing ions as a function of time. Over the course of a reaction, products may be formed in one region of the crystal and migrate to another—depending on the mass-to-charge ratios of the reactant and product ions. The rate at which this ion migration occurs is currently not well-known. As such, the technique introduced in this work has the potential to contribute to new measurements of the properties of trapped ions, as both the spatial and temporal distributions of all species that reach the detector can be recorded simultaneously.

## Methods

### Experimental Section

Coulomb crystals are formed within a linear Paul ion trap, following the same approach as outlined in previous work.^10−12^ A Ca oven is resistively heated, emitting Ca atoms that are subsequently nonresonantly ionized at the trap center by the 355 nm output of a frequency-tripled Nd:YAG laser. The Ca^+^ ions are Doppler cooled using 397 and 866 nm diodes addressing the 4s^2^S_1/2_ → 4p^2^P_1/2_ and 3d^2^D_3/2_ → 4p^2^P_1/2_ transitions, respectively. Coulomb crystals are formed once sufficient kinetic energy has been removed from the ions, resulting in a 3D lattice-like ellipsoid structure where the Coulombic repulsion between neighboring ions and the trapping forces confining the ions are balanced. The structure of the resultant Coulomb crystal is dependent on the amplitude of the radiofrequency trapping fields (*V*_rf_), drive frequency (Ω_rf_), and the static voltages (*V*_end_) applied to the trap end-caps. The Coulomb crystals formed as part of this work typically contain up to several hundred ions, with the fluorescence emitted as part of the laser cooling cycle detected using a CCD camera and 10× microscope objective lens.

Multicomponent Coulomb crystals are formed by admitting Kr gas into the chamber through a high-precision leak valve, with Kr^+^ formed *via* a [2 + 1] resonance-enhanced multiphoton ionization (REMPI) scheme using the 212 nm output of a Nd:YAG-pumped frequency-tripled dye laser.^10,11^ Kr^+^ ions are sympathetically cooled by elastic collisions with cotrapped laser-cooled Ca^+^ ions, yielding bicomponent Coulomb crystals. While only the Ca^+^ ions can be directly observed with the CCD camera, as fluorescence is only emitted by the laser-cooled species, the presence of other cotrapped species can be inferred from changes to the positions of Ca^+^ ions within the crystal structure.

When Kr^+^ ions are introduced to the crystal, they form an outer shell around the fluorescing Ca^+^ ions, with the Ca^+^ ions adopting positions closer to the trap axis and the crystal appearing to elongate. Once formed, Kr^+^ ions can undergo charge transfer reactions with background H_2_O in the chamber, forming H_2_O^+^ product ions.^10^ As the H_2_O^+^ ions have a lower mass-to-charge (*m*/*z*) ratio than both Kr^+^ and Ca^+^, they form a dark core along the central trap axis that can be observed in the CCD images. The H_2_O^+^ product ions can react further with background species in the chamber, abstracting a hydrogen atom and forming H_3_O^+^. As both primary H_2_O^+^ and secondary H_3_O^+^ product ions are present in some crystals, the separation of these adjacent peaks can be used to confirm that species differing in mass by 1 u are fully resolved.

At a selected time, ions are ejected from the trap and travel through a field-free flight tube and onto MCPs coupled with a phosphor screen (Photek VID 225, P47). To eject the ions, the RF trapping fields are switched off and damped prior to the application of static repeller and extractor fields to each pair of trap rods (as detailed previously)^24^—with a phosphor screen added to the detection setup for this set of measurements. All ions within the Coulomb crystal at the time of ejection are accelerated by the repeller and extractor fields until they reach a grounded mesh and pass into the field-free flight tube.

**Figure 1:**
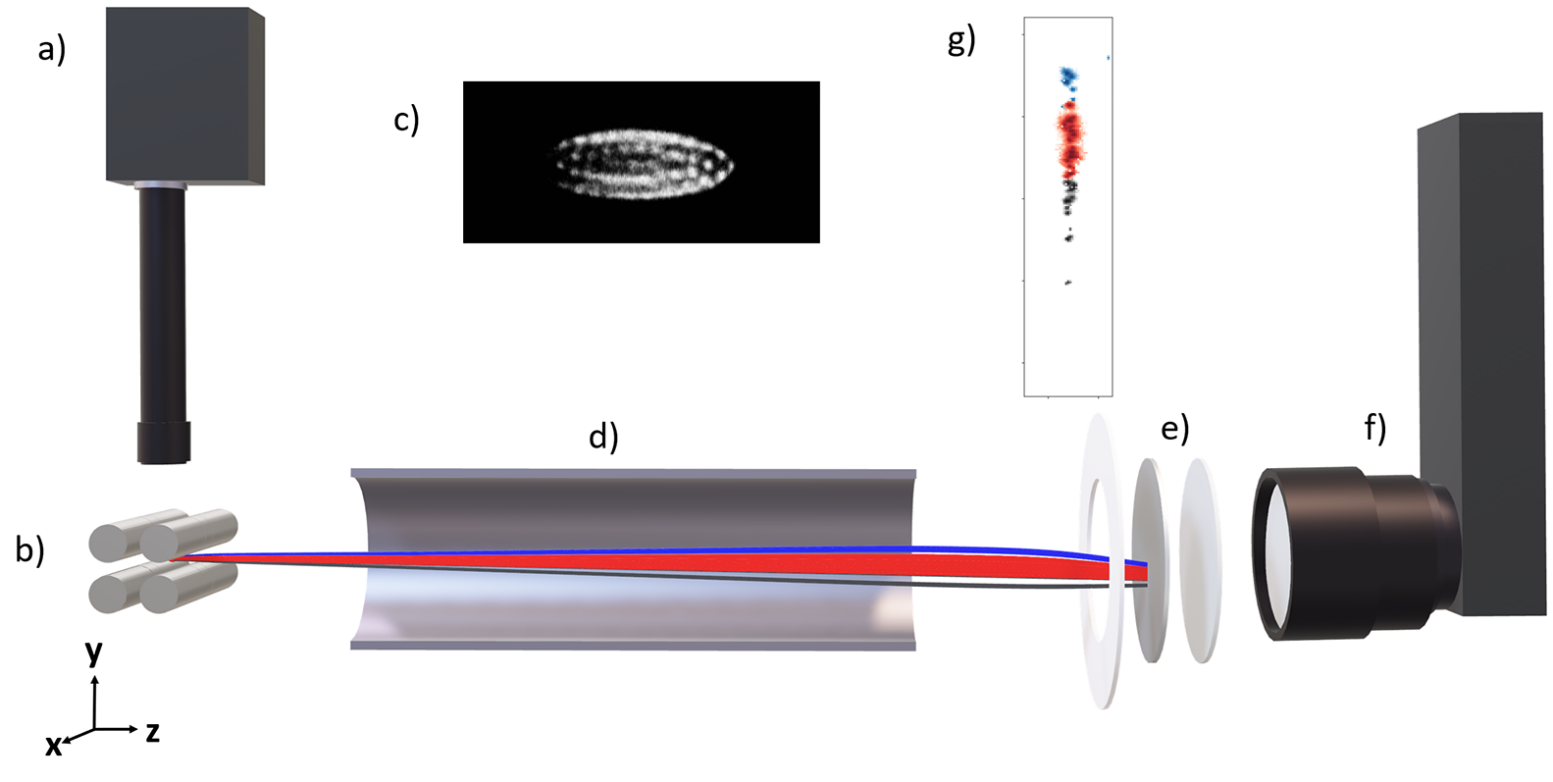
Schematic illustration (not to scale) depicting (a) CCD camera and 10× microscope lens, (b) linear Paul trap, (c) experimental fluorescence image of the multicomponent Coulomb crystal recorded by the CCD camera (containing 5 H_2_O^+^, 110 Ca^+^, and 5 Kr^+^ ions), (d) flight tube and SIMION trajectories of the ions ejected from the trap, (e) with the ions ultimately striking the MCPs and phosphor screen detector, (f) Timepix3 camera and objective lens (Irix 150 mm *f*/2.8 macro), and (g) false color Timepix3 signal of the ejected crystal observed on the phosphor screen, with H_2_O^+^ in blue, Ca^+^ in red, and Kr^+^ in gray.

**Figure 2:**
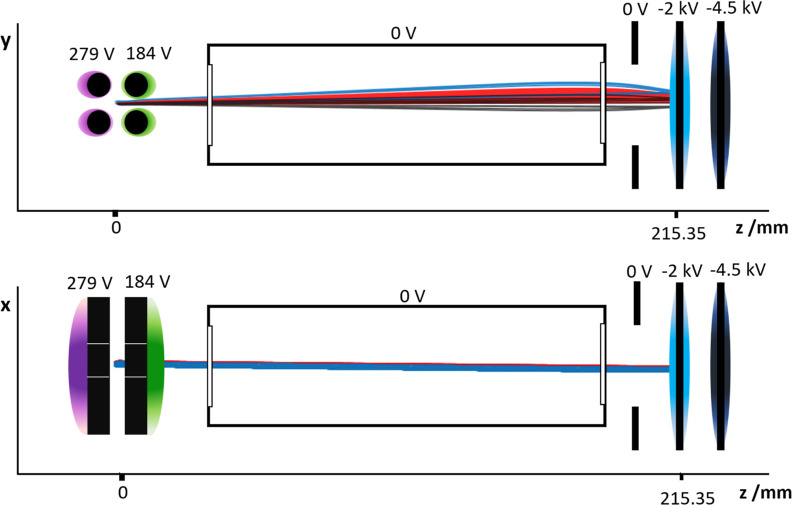
Schematic illustration of the ion trajectories and static fields present in the trap, flight tube, and detector during the ejection of ions. Top: *yz* view, bottom: *xz* view. As in Figure 1, the trajectories are those of a tricomponent crystal comprised of 5 H_2_O^+^ ions, 110 Ca^+^ ions, and 5 Kr^+^ ions. The trajectories of H_2_O^+^, Ca^+^, and Kr^+^ are shown in blue, red, and gray, respectively.

Ions of different *m*/*z* ratios are accelerated to different final velocities before entering the field-free region and so separate temporally during free flight. Ion arrival times at the detector can be accurately predicted by consideration of the Coulomb crystal properties, the distance between the trap and the detector, and the trapping and ejection fields that are applied to the trap rods. Phosphorescence is emitted when the ions hit the phosphor screen detector, with the positions, times, and intensities of this signal recorded using a Timepix3 camera. A schematic illustration of the experiment is given in Figures 1 and 2.

**Figure 3:**
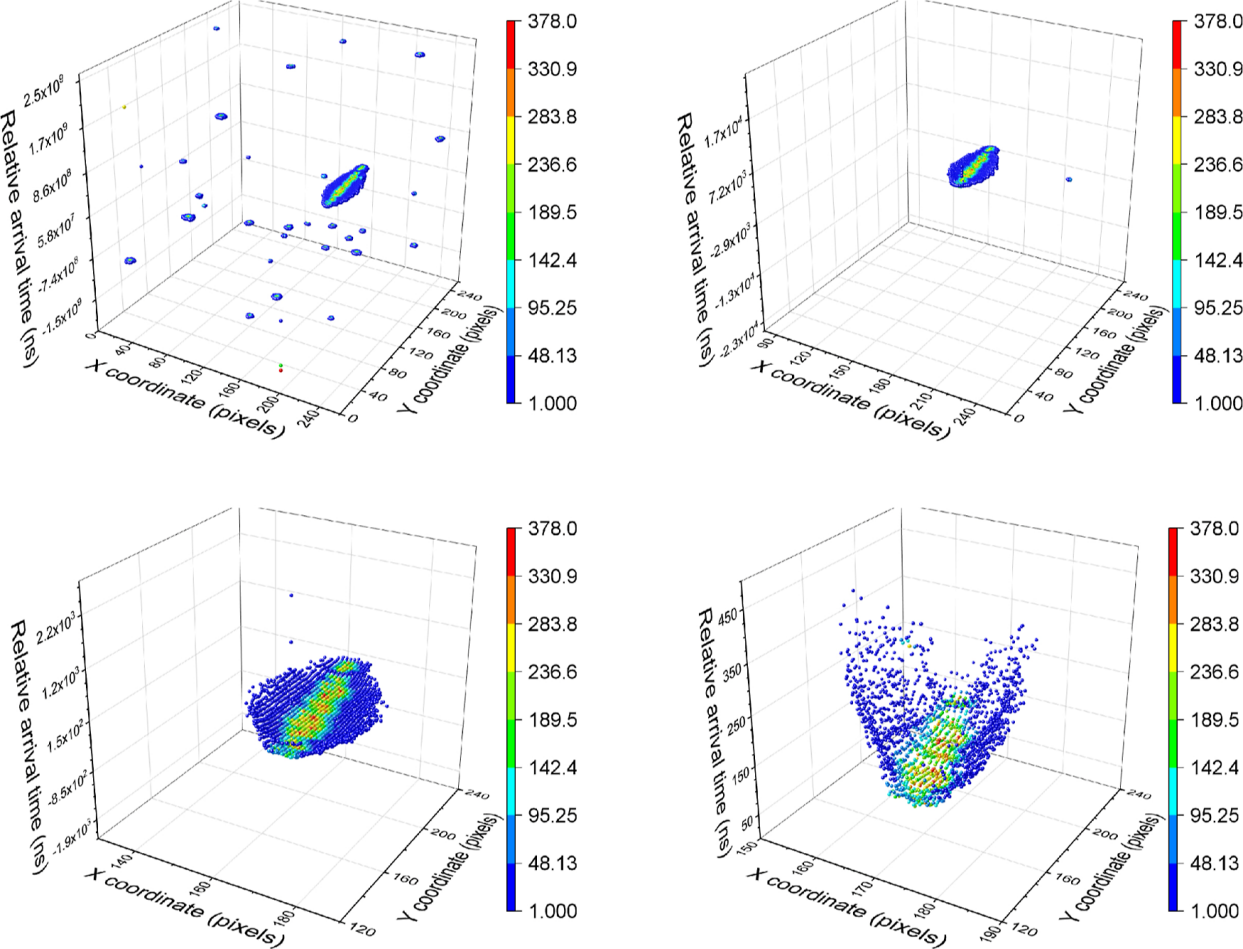
3D (*x*, *y*, and ToA) plots of a representative Coulomb crystal recorded using Timepix3. The ToA has been normalized so that zero corresponds to the arrival time of the Ca^+^ peak. The different time scales shown (top left: 5 × 10^9^ ns, top right: 5 × 10^4^ ns, bottom left: 5000 ns, and bottom right: 500 ns) demonstrate the high resolution possible with this approach. Cluster intensities (in ADU) are represented by the color scale. The “V” shape of the arrival time distribution in the bottom right plot arises from time-walk effects, as described in the text.

The Timepix3 application-specific integrated circuit (ASIC) consists of a 256 × 256 pixel array, with each pixel being a square of side length 55 μm.^32^ Every pixel within the camera has an independent readout, providing a camera with native zero suppression. When a pixel of the camera detects a signal above a predefined threshold, the readout process is triggered. The time at which the signal went over the threshold is known as the ToA and is recorded with a 1.6 ns resolution. The duration in time for which this signal remains over the threshold is known as the ToT, used as a proxy for intensity, and is recorded with 10 bit resolution. In this application, the 256 × 256 pixel array of Timepix3 allows the camera to detect simultaneous cluster position distribution information (pixel location), ToF information (ToA in ns relative to ejection), and cluster intensity [ToT in analog-to-digital units (ADU)]. The combination of these three quantities within a single device provides powerful measurement capabilities. The data produced by the camera is a stream of hits containing *x*, *y*, ToA, and ToT information, with the distribution in *x*, *y*, and ToA for a representative monocomponent crystal shown in Figure 3.

In this work, the Timepix3 data is typically visualized either as a 2D image, by integrating the (*x*, *y*) signal over a range of ToA, or as summed intensity (in the *xy* plane) as a function of ToA. The former allows for direct comparison with the simulated distributions extracted from the SIMION simulations (detailed below), while the latter can be used to determine the chemical composition of a given crystal. For the 2D images, a rotation of 6° is applied to correct for an offset in the mounting of the Timepix3 camera with respect to the *y* (vertical) axis. (The small white dots that can be seen in the rotated images are artifacts of the coordinate transformation and do not affect the data analysis.) Full details on both methods of data treatment are given in the Supporting Information.

An effect known as “time walk” is often observed in fast-response camera systems such as Timepix3. For signals that are known to be synchronous, the camera may detect later arrival times for lower-intensity signals compared to higher-intensity signals. This effect is explained by the specific response characteristics of the in-pixel amplifiers and thresholding process.^49,50^ The relative error introduced by these time-walk effects is not significant for the results presented in this work, and so no time-walk correction is performed. To illustrate the effect, a representative Ca^+^ peak is shown in Figure S4 in the Supporting Information. A mass resolution of *m*/Δ*m* ≈ 350 is calculated at *m*/*z* = 40, confirming that the technique is capable of resolving species that differ in mass by 1 u over the *m*/*z* range of interest in this work. The mass resolution is further evidenced by the detection of distinct H_2_O^+^ and H_3_O^+^ peaks (see Supporting Information for further details).

### SIMION Analysis

Ion trajectories are modeled using SIMION,^51^ where velocity-Verlet algorithms are integrated to solve the equations of motion of the ions as they travel through different regions of the apparatus. A computer-aided design (CAD) model of the experimental apparatus was created in Autodesk Inventor and imported into SIMION, where time-dependent electrostatic fields can be specified to match the relevant experimental conditions. In order to accurately represent the fields experienced by the ions, experimentally measured trapping and ejection fields are applied to the ion trap rods in SIMION. The importance of including the nonideal experimental fields is detailed in the Results section.

The numbers, identities, and properties of the ions within the trap are determined by CCMD simulations based on the images of the crystal captured by the CCD camera immediately prior to ejection. CCMD simulations have been described previously,^11,24^ with further details provided in the Supporting Information. The initial positions and velocities of each ion, established from the CCMD simulations, are imported into SIMION and subsequently propagated in time through the different regions of the apparatus. From this information, it is possible to simulate the flight time, trajectory, and impact position of every ion within the Coulomb crystal—facilitating a direct comparison with the experimental measurements.

## Results

### Monocomponent Crystals

**Figure 4:**
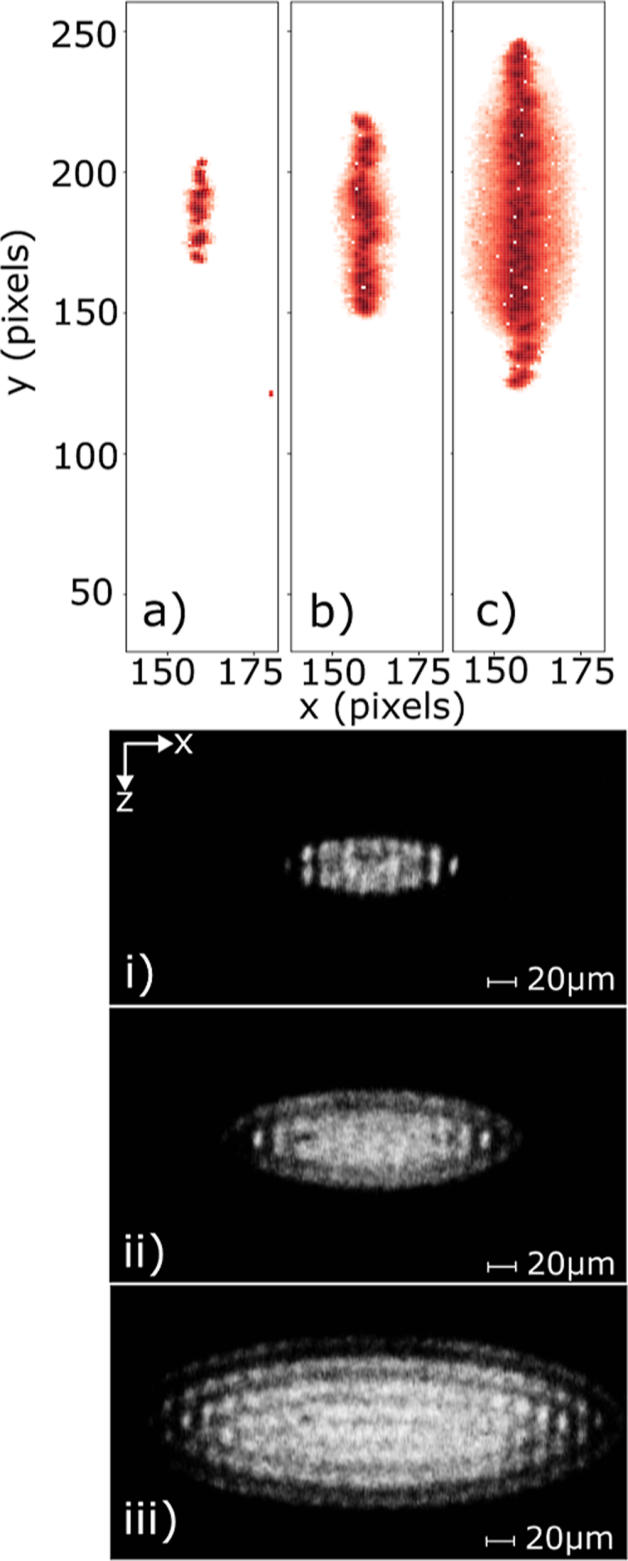
Three different sizes of Ca^+^ Coulomb crystal are shown, as imaged by the CCD camera prior to ejection (bottom) with the corresponding phosphor screen signals detected by the Timepix3 camera output (top). Axis orientations are given relative to the lab frame, as illustrated in the 3D schematic shown in Figure 1. Plots (a,i) contain 30, (b,ii) 130, and (c,iii) 565 ions. The color map indicates the intensity of the photons on the phosphor screen, with the darker red representing a more intense signal.

A clear correlation can be seen between the number of Ca^+^ ions in a Coulomb crystal and the physical size of the crystal observed by the two complementary camera systems. Figure 4 shows three monocomponent Coulomb crystals, displaying the CCD camera images of the Coulomb crystals prior to ejection (below) and the corresponding time-selected signals recorded by the Timepix3 camera at the phosphor screen after ejection (above). The spatial distribution of the ToF peaks can be recorded with exceptional resolution (see Figure 3); further details on the Timepix3 data are given in the Supporting Information.

As illustrated schematically in Figure 1 and shown in the images presented in Figure 4, ions within the Coulomb crystal are arranged such that the central axis of the ellipsoid lies along the trap (*x*) axis. Repeller and extractor voltages are applied to the four cylindrical trap rods—giving rise to an ejection field that is less uniform than the fields generated by planar repeller and extractor electrodes.^24^ As a consequence of the asymmetry in the fields, the ejected ion cloud exhibits a spatial distribution along the *y*-axis (*i.e.*, perpendicular to both the trap and ToF axes) that is larger than that along the *x*-axis (the trapping axis), with this effect shown in Figure 4. Note that the experimental phosphor screen image is rotated by 6° to account for a tilt in the mounting of the Timepix3 camera. Quantitative agreement is observed between the ion numbers of the crystal (as determined by CCMD simulations) and the integrated Timepix3 signal for crystals containing up to 600 Ca^+^ ions. From the probability of multiple ion strikes on a single MCP channel (determined by the SIMION simulations) and the transmission efficiency of the flight tube mesh,^52^ a cumulative detection efficiency of 83% is obtained for crystals containing 300 or fewer Ca^+^ ions. Further details are given in the Supporting Information.

**Figure 5:**
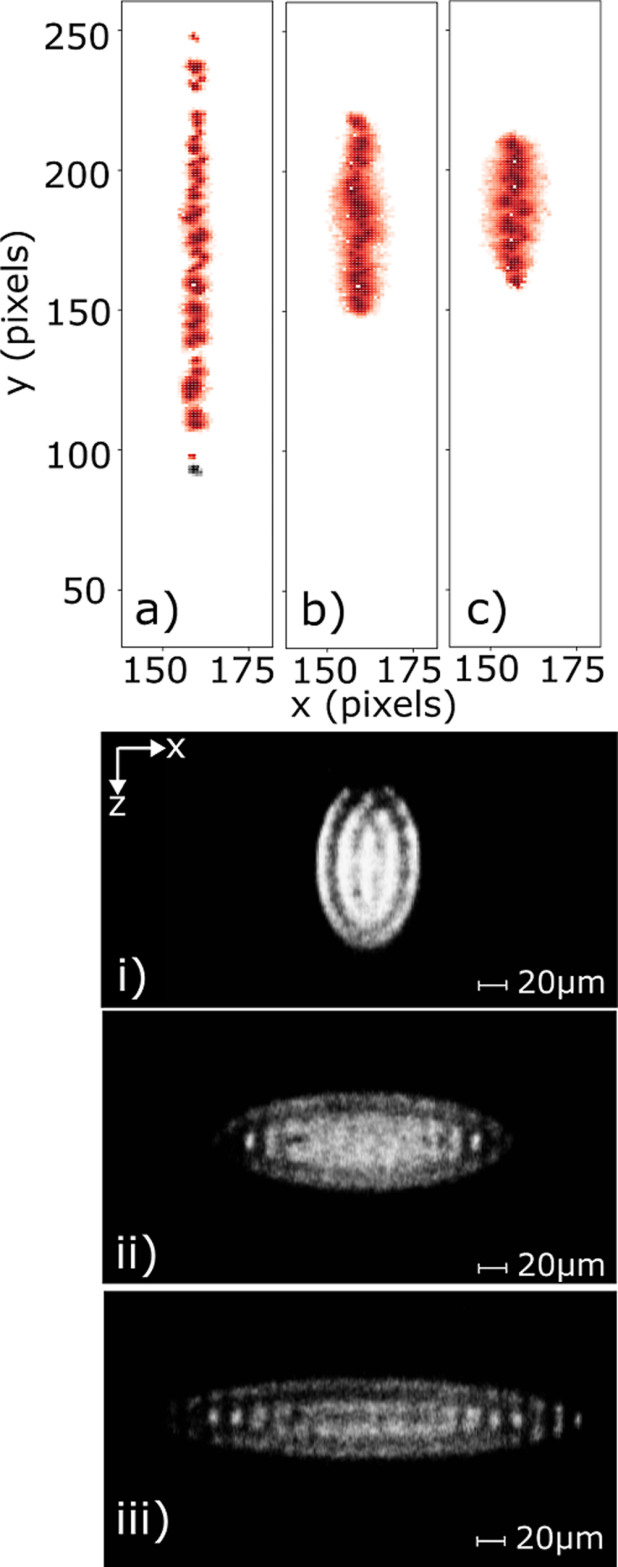
Coulomb crystal fluorescence images recorded by the CCD camera (bottom) and the corresponding phosphor screen signals detected by the Timepix3 camera output (top) are shown. Axis orientations are given relative to the lab frame, as illustrated in the 3D schematic shown in Figure 1. In each instance, approximately 130 Ca^+^ ions are confined at a series of different end-cap voltages: (a,i) 7.86, (b,ii) 2.23, and (c,iii) 1.13 V. The color map indicates the intensity of the photons on the phosphor screen, with the darker red representing a more intense signal.

To further explore the relationship between the Ca^+^ spatial distribution within the crystal and the distribution observed at the phosphor screen following ejection, the end-cap voltage (*V*_end_) and the amplitude of the RF trapping fields (*V*_rf_) can be varied. A direct correlation can be observed between the crystal geometry recorded in the CCD images and the ejected ion signal measured on the phosphor screen. As shown in Figure 5, increasing *V*_end_ causes the crystal to adopt a more prolate structure in the ion trap (instead of the typical oblate arrangement), making the crystal less elongated along the trap axis.

A similar trend is observed when the amplitude of the RF trapping field is altered, with greater *V*_rf_ elongating the crystal along the trap axis (*i.e.*, forming more oblate crystals) associated with a reduced *y*-axis distribution at the phosphor screen when compared to (more spherical) crystals formed with a lower *V*_rf_. In all cases, the experimental distributions observed at the phosphor screen can be accurately reproduced by SIMION trajectory simulations—as the geometry of the electrodes, the properties of the ions within the crystal at the point of ejection, and the experimental ejection fields are included as SIMION input parameters.

### Multicomponent Crystals

Laser-cooled ions can be straightforwardly monitored by using a CCD camera. However, it is not possible to directly image the “dark” (nonlaser-cooled) ions; only the laser-cooled species emit fluorescence. It is possible to detect nonfluorescing species by (for example) ejecting the crystal and recording ToF traces, but no structural information is retained about the positions of the dark ions within the crystal.

**Figure 6:**
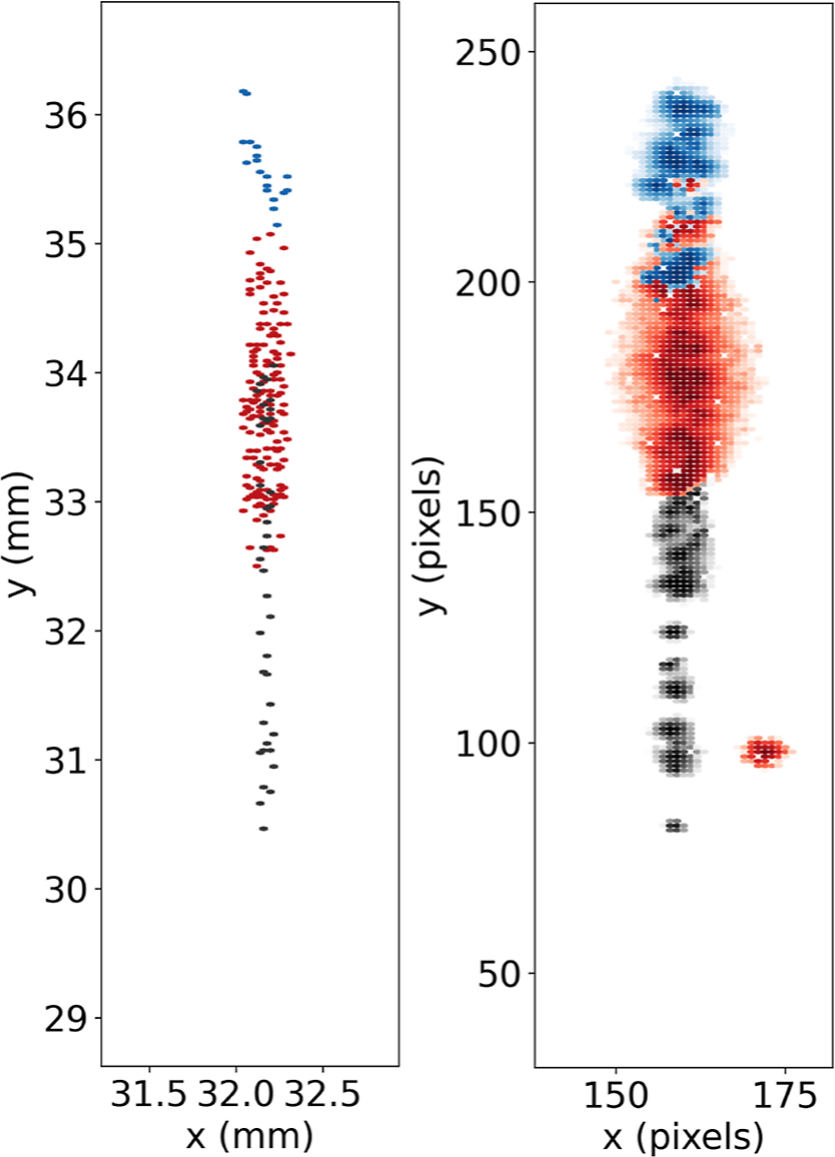
Comparison between the spatial distribution of ions at the phosphor screen detector as observed by the Timepix3 camera, integrated over time (right), and from the SIMION simulation of the ejected crystal (left). The different ions within the multicomponent crystal (H_2_O^+^, Ca^+^, and Kr^+^) are indicated by different colors (blue, red, and gray points, respectively). The red ion strike in the bottom right of the experimental plot can be attributed to background noise detected in the same time window as the Ca^+^ ions.

If the identities of the nonfluorescing ions are known, then the composition of the crystal can be established by comparing the experimental crystal images (from the CCD camera) with CCMD simulations. The resulting simulated crystals are then used as inputs for the SIMION simulations, allowing both the trajectories of the ions through the ToF tube and their spatial and temporal distributions at the phosphor screen to be calculated. A simulated spatial distribution of ions at the detector is presented in Figure 6, alongside the experimental distribution observed by the Timepix3 camera for the corresponding crystal. The excellent agreement between the two plots confirms that the relative numbers and positions of the dark ions in the simulated crystals are accurately described.

Interestingly, analysis of the multicomponent crystals following ejection onto the phosphor screen reveals a distinct spatial distribution; different ionic species strike the detector centered around different points along the *y*-axis, as shown in Figure 6. As no prior Coulomb crystal ejection studies have employed a phosphor screen with a fast-response camera (such as the Timepix3 used in this work), there are no prior measurements of the spatial distribution of ions as they strike the MCPs, requiring careful analysis of this effect.

**Figure 7:**
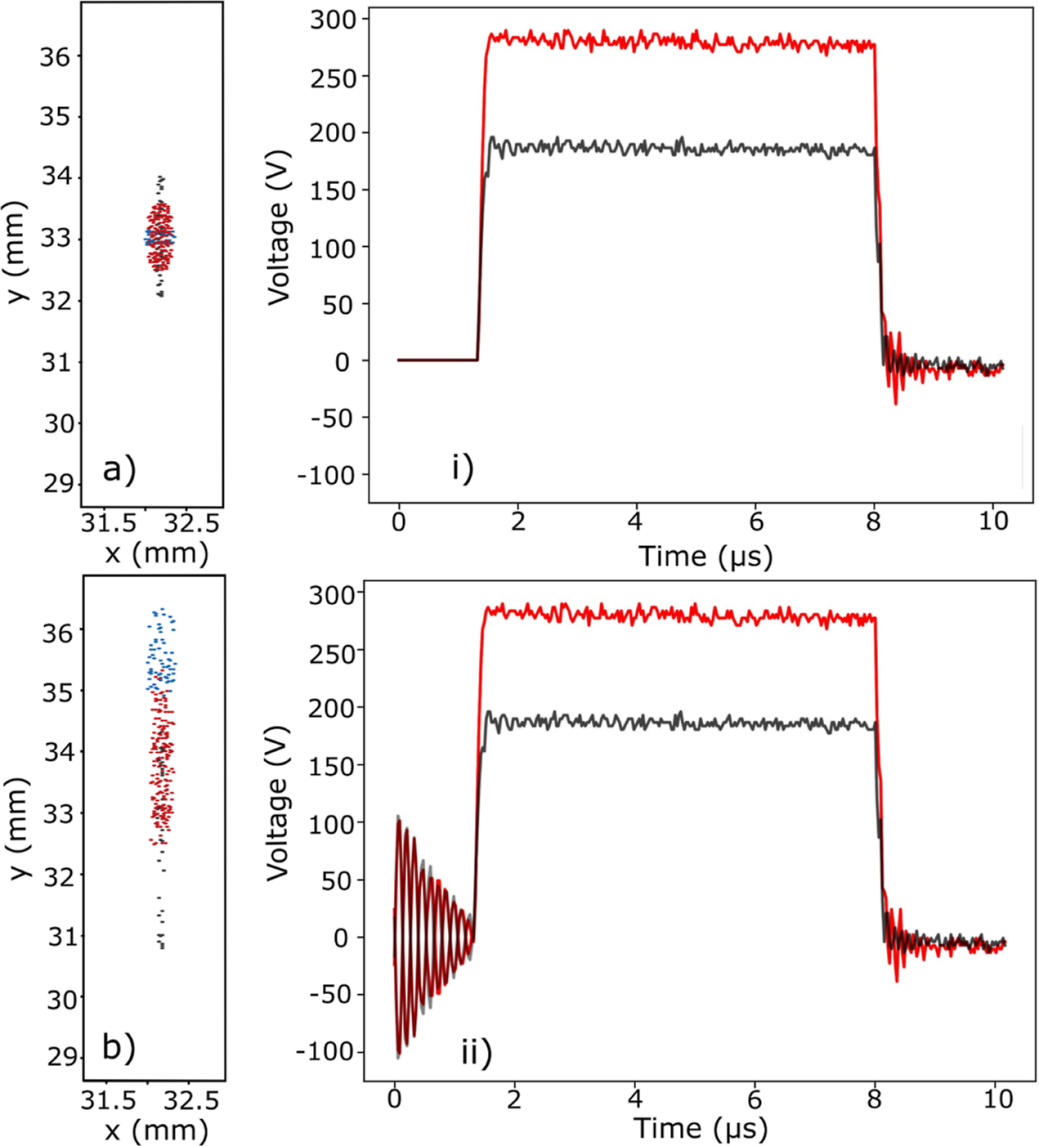
Calculated spatial distribution of the ejected ions at the detector is shown in (a,b), with the different ions (H_2_O^+^, Ca^+^, and Kr^+^) shown in different colors (blue, red, and gray points, respectively). The corresponding time-dependent ejection waveforms applied in SIMION are shown on the right, (i,ii). The experimental repeller and extractor fields (ii) generate the distribution shown in (b), whereas ejection fields with no ringing noise present (i) give rise to the distribution depicted in (a).

One potential source of the spatial separation of the different ionic species is the nonzero decay time and nonideality of the trapping fields prior to the application of the extraction fields. Experimentally, the sinusoidal RF trapping fields decay for approximately 1.3 μs before static repeller and extractor fields are applied. The experimental fields exhibit some imperfections, as can be seen in the minor differences between the amplitudes of the repeller (red) and extractor (black) traces over the 1.3 μs decay of the RF fields. Finally, it is also possible that the center of the pseudopotential generated by the trapping fields is not precisely in the geometric center of the trap. By including these effects in the SIMION simulations, it is possible to recreate the experimentally observed *m*/*z*-dependent spatial separation at the phosphor screen. When the experimental repeller and extractor fields are applied without the decaying RF trapping fields (*i.e.*, with no ringing immediately prior to the ejection pulses in the time-dependent waveforms) and with the crystal located in the center of the trap, no *m*/*z*-dependent spatial separation of the signal is observed at the phosphor screen, as can be seen in Figure 7 (*i.e.*, the ion strikes for different ionic species are concentric).

The observed spatial separation could also arise from a misalignment between the trap center and the ToF detection setup or from the presence of stray fields. While the simulated distribution of ions at the phosphor screen does show a dependence on some of these effects, it cannot reproduce the experimental results. The effects with the most influence are the offset of the crystal from the geometric center of the trap and the inclusion of experimental trapping, damping, and ejection fields. Only when the experimental trapping, damping, and ejection fields are included—alongside a minor offset of the crystal (approximately 0.4 mm in *z*) from the geometric trap center—can the experimentally observed spatial distribution be reproduced in the simulations. This observation not only allows for the rationalization of the observed experimental data but also highlights the importance of including as many experimental details as possible in the ion trajectory simulations.

Finally, SIMION simulations also allow for the determination of the so-called “double hit” rate—the probability that two ions strike the same channel on the MCPs within a short time frame, thereby leading to the nonobservation of the second ion. For Coulomb crystals containing up to several hundred total ions, the predicted double hit rate is low for both mono- and multicomponent crystals, suggesting that this is not a significant factor under our experimental conditions. This conclusion is reinforced by the strong correlation between the ion numbers assigned to the recorded CCD images and the measured MCP signal intensity, as detailed in the previous subsection and discussed in the Supporting Information.

Using the apparatus employed in this study, it is challenging to quantify the detection efficiency of all ions within multicomponent crystals—although some details are provided in the Supporting Information. With the incorporation of a longer flight tube and the collection of additional experimental data, it is expected that a more quantitative approach will be possible in the future.

## Discussion and Conclusions

In this work, we report the first direct experimental measurements of the positions of dark, nonfluorescing ions within Coulomb crystals at the point of ejection. This has been achieved by the addition of a phosphor screen and Timepix3 camera to the MCP detector at the end of a flight tube, allowing for the simultaneous recording of ion positions and flight times. While positional information has previously been inferred from matching experimental Coulomb crystal images with simulated CCMD images, this required assumptions to be made and the accuracy of simulated multicomponent Coulomb crystal structures could not be independently verified.

To rationalize the observed temporal and spatial distributions recorded at the phosphor screen, Coulomb crystal properties (from CCMD simulations) are incorporated into SIMION, where the experimental apparatus is described and experimental ejection waveforms are applied to the trap electrodes. The trajectories of the ejected ions are established as they fly through the various regions of the apparatus, ultimately reaching the MCPs and the phosphor screen detector. The spatial distribution of the simulated ion signal on the phosphor screen can be compared directly to the experimental distributions recorded with the Timepix3 camera.

Excellent agreement is seen between the simulated and measured spatial distributions of the ion strikes on the phosphor screen. In particular, the experimentally observed *m*/*z*-dependence of the spatial distribution is reproduced in the simulated images. The experimental results could only be accounted for when the real ejection waveforms (including the decaying nonideal sinusoidal RF trapping fields present prior to the application of static repeller and extractor fields) and a small offset in the crystal position from the geometric trap center were explicitly included in the simulations. Our findings highlight the importance of describing all experimental parameters as accurately as possible when conducting detailed simulations. The agreement between the experimental Timepix3 measurements and the simulated SIMION distributions provides independent validation that the CCMD simulations can accurately capture the positions of all ions within multicomponent crystals.

It is anticipated that the combination of spatial and temporal detection capabilities will facilitate a range of new measurements in the near future. For example, it may be possible to determine important experimental parameters, such as the rates of sympathetic cooling, and to map ion migration through the crystal. Akin to what is seen in the velocity map imaging technique,^27,54^ SIMION simulations show that the spatial distribution of the ion signal at the detector increases with increasing ion kinetic energy, especially along the *y*-axis. The simulated spatial distribution at the detector is also sensitive to the positions of the ions within the crystal at the point of ejection. Through recording a series of precisely timed measurements, alongside CCMD and SIMION simulations, the velocities and positions of sympathetically cooled ions could be established as a function of time. Undertaking these measurements will require some amendments to the current experimental setup and will therefore be the subject of future investigations.

As the results presented here demonstrate, the spatial distribution of the ion signal at the phosphor screen is highly dependent on the trap parameters and properties of the ions within the crystal. While further work is required to determine the full capabilities of the method, the high sensitivity to the positions, identities, and energies of the ions could lead to new measurements of time-dependent processes within Coulomb crystals—going beyond what conventional ToF-MS measurements and CCMD simulations can provide.

## Data Availability

Supporting data can be obtained from DataCat, the University of Liverpool Research Data Catalogue, at https://doi.org/10.17638/datacat.liverpool.ac.uk/2633.

## Supporting Information Available

Data analysis, temporal and mass resolution of the method, quantification of the phosphor screen signal, and sensitivity to the ion positions within the trap (PDF)
