# Supplementary material for: Spatial and Temporal Detection of Ions Ejected from Coulomb Crystals
Source: J Phys Chem A. 2024 Apr 8;128(19):3900–9. doi: 10.1021/acs.jpca.3c08132 (PMC11103685; doi:10.1021/acs.jpca.3c08132)
Supplement: Supplementary file 1 — jp3c08132_si_001.pdf [file jp3c08132_si_001.pdf]

Supporting Information for Publication: Spatial and Temporal Detection of Ions Ejected from Coulomb Crystals

Jake A. Diprose,[†] Vincent Richardson,[†] Paul Regan,[†] Adam Roberts,[†] Sergey Burdin,[†] Andriana Tsikritea,[†,‡] Konstantinos Mavrokoridis,[†] and Brianna R. Heazlewood[*,†]

†*Department of Physics, University of Liverpool, Liverpool, L69 7ZE, United Kingdom*

‡*Department of Physics, TU Dortmund, 44227 Dortmund, Germany*

E-mail: b.r.heazlewood@liverpool.ac.uk

Data Analysis

Data recorded using the Timepix3 camera is in the form of time-over-threshold (ToT) signal, which acts as a proxy for the intensity (I) of ion strikes at a given 2D position (x, y), and time-of-arrival (ToA) signal. In order to avoid degradation of the phosphor screen and limit the size of the data files, the Timepix3 exposure is limited to a ∼5 s window spanning the time of ejection. The recorded ToT signal is binned in 1 ns time steps. The time-of-flight of the ion signal is defined with respect to the arrival of the Ca^+ peak, which has been previously measured in this apparatus to be 6400 ns (with 0 ns defined as the time that the ejection fields are applied).

Plots of the ion strikes on the detector can be generated by integrating the signal over a given time window on a pixel-by-pixel basis. In order to be consistent in the data analysis, the recorded ToA data is divided into five different time 'windows'—facilitating the separation of the contributions made by different ions within the crystal. The first and last time windows contain only spurious background ion strikes, as these identify regions before the ejection

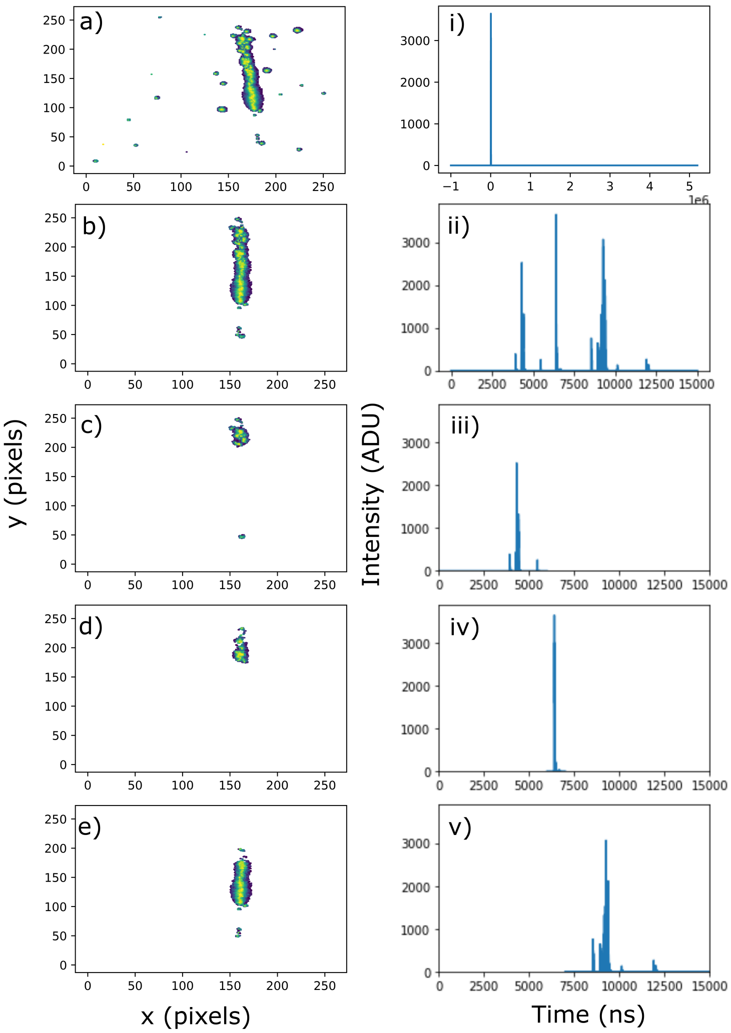

Figure S1: Time-gated x, y Timepix3 signal output is shown on the left [panels (a) to (e)], with intensity indicated by the colour map. The integrated 2D data is provided on the right, plotted as a function of time. The first pair of plots, (a) and (i), show the signal recorded over the entire time window. Plots (b) and (ii) show the signal recorded in the time window when the ejected ions from the Coulomb crystal reach the detector (i.e., excluding the spurious ion strikes that occur outside the detection window). Plots (c)-(e) and (iii)-(v) illustrate the ion strikes occurring within narrower time windows. Plots (c) and (iii) depict the light ions (H_2O^+/H_3O^+), (d) and (iv) show the Ca^+ ions, and plots (e) and (v) the heavy ions (Kr^+). Plot (a) shows the raw data from the Timepix3 camera, whilst plots (b)-(e) are adjusted to account for the 6° tilt in the mounting of camera system.

fields are applied and more than 15000 ns after ion ejection (corresponding to ions with m/z ratios beyond what can be incorporated into Coulomb crystals, given the ion trap operating parameters). The remaining 15000 ns window is then split into three: the arrival window for

lighter ions, present in the dark core of the crystal (0–6000 ns), the arrival window for Ca^+ ions (6000–7000 ns), and the arrival window for heavier ions (7000–15000 ns). The multi-component 2D distribution plots shown in the main text are then generated by integrating the signal at each pixel over each of these time windows. Each step of the data analysis is illustrated in Figure S1.

After installation, the Timepix3 camera was found to be mounted at an angle of 6° with respect to the vertical (y) axis. The Timepix3 experimental data is therefore rotated by 6°, to allow for direct comparison with SIMION simulations. As illustrated in Figure S2, small white dots can be seen periodically in the rotated image. The dots are blank pixels, and they arise due to the lack of one-to-one correspondence between the original and final pixel coordinates. The total signal intensity is maintained, and the quality of the original data is not compromised in the subsequent analysis; the coordinates are simply transformed.

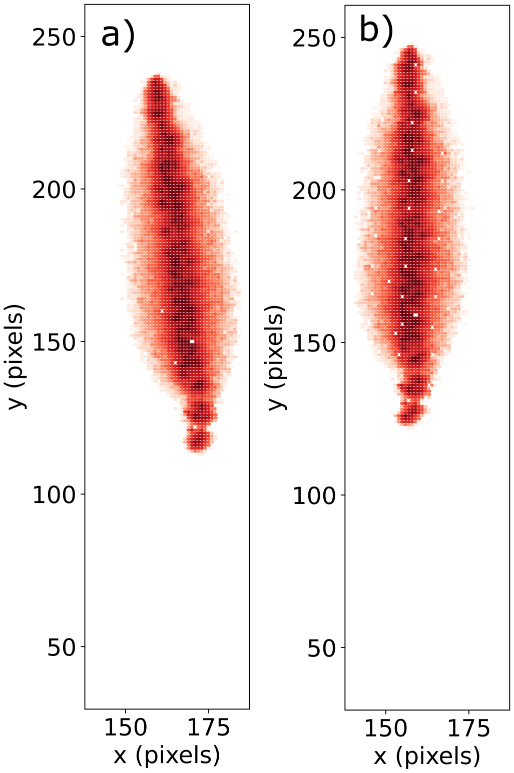

Figure S2: Time-gated x, y Timepix3 signal shown (a) as collected by the camera, and (b) after being rotated by 6°, to correct for the offset in the mounting of the camera.

Temporal and Mass Resolution

The resolution of the Timepix3 camera system as used in this project is 1.5625 ns. A P47 phosphor screen decay time of 80 ns has been provided by the supplier [1]. (It is noted that other phosphor screen suppliers state decay times of 400 ns to 1% (and 100 ns to 10%).[2]) As the flight times of the different ionic species involved in this work differ by μs, the response rates of the Timepix3 camera and phosphor screen are much faster than what is required for this application.

As discussed in the main text, it is possible for there to be multiple ion strikes hitting the same channel of the MCP detector, and the recovery time of the MCPs is challenging to quantify. If a channel has insufficient time to recover from the first ion strike when a second ion hits it, then the second ion strike will not be recorded. An example of this behaviour can be seen in Figure S3. When the Ca^+ signal is removed, it can be seen that the upper section of the (later-arriving) Kr^+ ion strikes in plot (a) are missing; the overall profile of the Kr^+ ion distribution can be seen, but there is intensity missing in the region between $y = 170 - 205$ pixels. From analysis of the fluorescence images (provided by CCMD simulations), this crystal contains approximately 520 ions—consistent with our prediction (from SIMION simulations) that double hits start to become more appreciable in multi-component crystals containing >500 ions. The Timepix3 camera data thus clearly illustrates the importance of considering double-hits in the analysis of time-of-flight data, to account for the reduced detection efficiency of heavier ions in large crystals.

Table S1: Ion numbers assigned to the Coulomb crystals corresponding to the Timepix3 plots given in Figure S3.

Crystal	H_2O^+	Ca^+	Kr^+
(a)	26	215	280
(b)	20	200	20
(c)	5	100	5

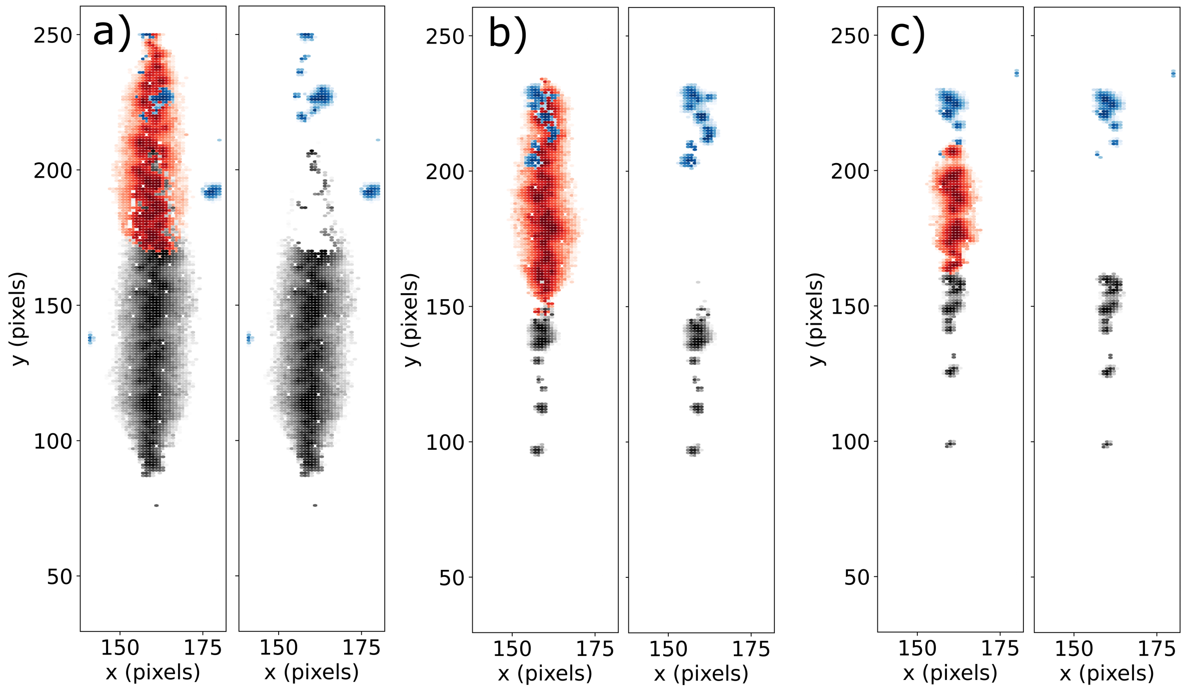

Figure S3: Experimental Timepix3 data showing the 2D distribution of ion strikes in 3 different Coulomb crystals of varying ion numbers. H_2O^+ are shown in blue, Ca^+ in red, and Kr^+ in black. The corresponding ion numbers are given in Table S1. Plots are provided in pairs: all ion strikes arriving in the pre-defined detection window are shown on the left, with the contribution of the Ca^+ signal removed in the plots on the right.

The experimental ToF traces can be converted into mass spectra, with the corresponding mass resolution defined in Equation S1, where m is the mass of a singly charged ion.

$$\text{Mass Resolution} = \frac{m}{\Delta m} \tag{S1}$$

As the peaks recorded using the Timepix3 camera are asymmetric, they cannot be fit to a Gaussian distribution. Instead, the two components of a given mass peak—the rising edge and the decaying tail—can be fit separately. This approach provides a method for reliably establishing the full-width-at-half-maximum (FWHM) of a given mass peak, and therefore the mass resolution. A representative Ca^+ peak is shown in Figure S4, where the two parts of the distribution are fit (separately) to double exponential functions. The sum of the two half lives established from the fits is equivalent to the FWHM of the peak, and a value of $\Delta(m) \approx 0.11$ u at $m/z = 40$ is calculated using this approach. An average mass resolution

of approximately 350 is determined for Ca^+ ($m/z = 40$) in this work. As mentioned in the main text, the mass resolution is further evidenced by the ability to observe distinct $m/z = 18$ (H_2O^+) and $m/z = 19$ (H_3O^+) peaks, as can be seen in Figure S5.

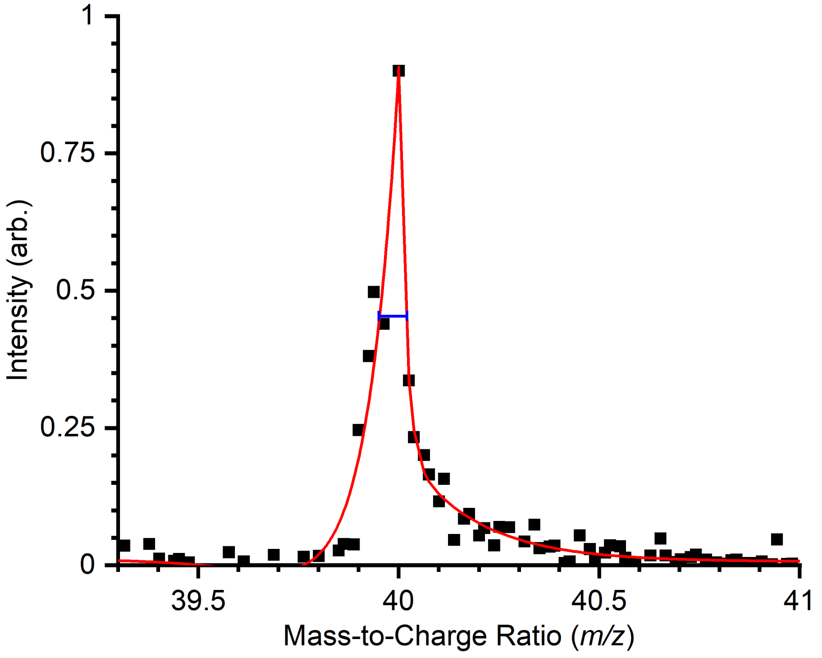

Figure S4: Plot of intensity as a function of m/z for a representative Ca^+ mass peak recorded using the Timepix3 camera. The experimental signal is shown as black squares, with fits to the data provided in red. The rising and falling components of the peak are fit separately, enabling the FWHM of the distribution to be established (indicated by a horizontal blue line).

Quantifying the Phosphor Screen Signal

To extract quantitative information on the composition of a given Coulomb crystal from the Timepix3 data, the relationship between the signal recorded by the Timepix3 camera and the number of ions in the crystal needs to be quantified. A comparison is therefore made between the Timepix3 data and the ion numbers assigned to the experimental crystals using CCMD simulations—a well-established method adopted in previous studies and outlined in

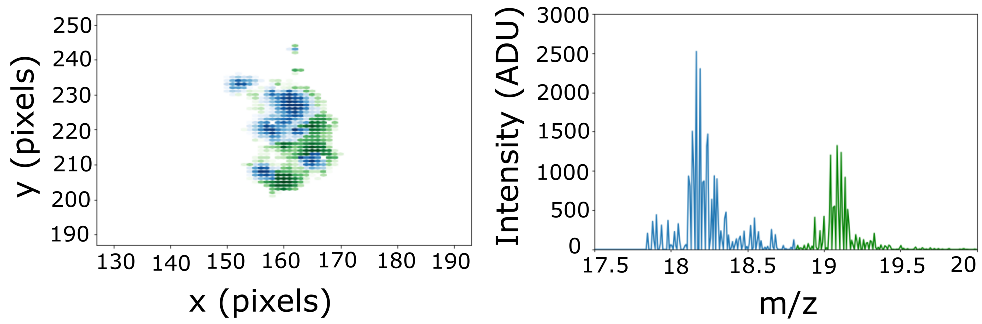

Figure S5: Data presented in Figure S1, panels (c) and (iii), is provided here to show the contributions of H_2O^+ (blue) and H_3O^+ (green) ions more clearly. Time-gated x, y Timepix3 signal output is shown on the left, with the integrated 2D data provided on the right, illustrating the separation of the $m/z = 18$ (H_2O^+) and $m/z = 19$ (H_3O^+) peaks.

the main text. A multi-component crystal with a large percentage of non-laser-cooled ions is provided in Figure S6, where the experimental fluorescence image collected by the CCD camera is shown alongside the corresponding CCMD simulation.

The correlation between the integrated Ca^+ peak from the Timepix3 data [reported in Analogue to Digital Units (ADU)] and the assigned ion numbers using CCMD is shown in Figure S7. As a clear linear relationship exists between the Timepix3 signal and the number of ions with a given Coulomb crystal, the relationship set out in Equation S2 can be extracted.

$$\text{Intensity (ADU)} = \text{Number of ions} \times 419.2 \pm 4.9 \qquad \text{(S2)}$$

Equation S2 quantifies the relationship between the signal recorded by the Timepix3 camera-based detection system and the number of Ca^+ ions in the original Coulomb crystal. For Ca^+ crystals containing fewer than 600 ions, we obtain an R^2 value of 0.99 when fitting a straight line to the data, demonstrating the ability of the Timepix3 camera to provide reliable and quantitative information on the composition of Coulomb crystals. It should be noted that this linear fit only holds for crystals containing fewer than 600 ions. For mono-component crystals containing more than 600 ions, or multi-component crystals with > 500

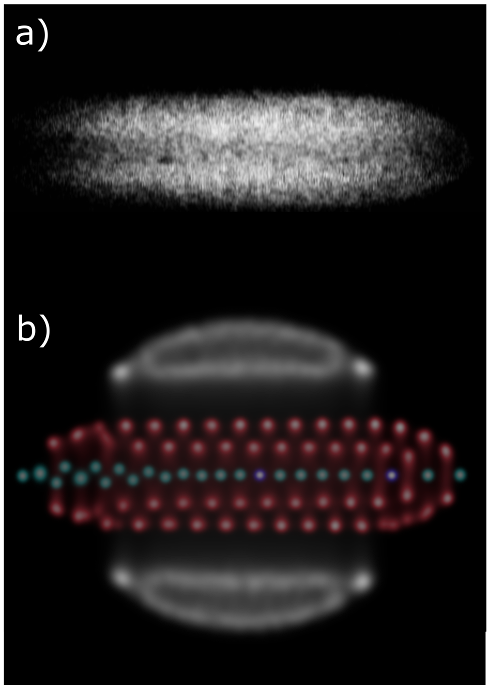

Figure S6: Comparison of (a) an experimental CCD image of the fluorescence emitted by Ca^+ ions in the crystal, and (b) the corresponding (false colour) simulated CCMD crystal, where the different ionic species present are indicated by colour. The laser-cooled Ca^+ ions—the only species that can be observed directly by the camera—are red, the Kr^+ ions are gray, and the H_2O^+ and H_3O^+ ions are blue. As the positions of the H_2O^+ and H_3O^+ ions are indistinguishable in the CCMD simulations (both species reside in the dark core and frequently exchange positions), the contribution of each channel is established using the ToF data.

ions, the number of 'double strikes' (shown in Figure S8) starts to exceed 10% of the total ions in the crystal and is therefore not considered in this analysis.

Double strikes are deemed to occur in the simulations when two or more ions hit the detector within the same spatial region (i.e., are separated by 12 μm or less). The distance of 12 μm corresponds to the diameter of the MCP pores; ions separated by more than 12 μm should not impinge on a single pore, whereas ions separated by less than 12 μm could both hit the same pore. As such, the number of double strikes identified within the SIMION simulations is likely to overestimate the true double strike probability, as a pair of ions will sometimes hit neighbouring pores when separated by < 12 μm. As the double hit rate is included in the detection efficiency calculations, a cautious approach is adopted here to

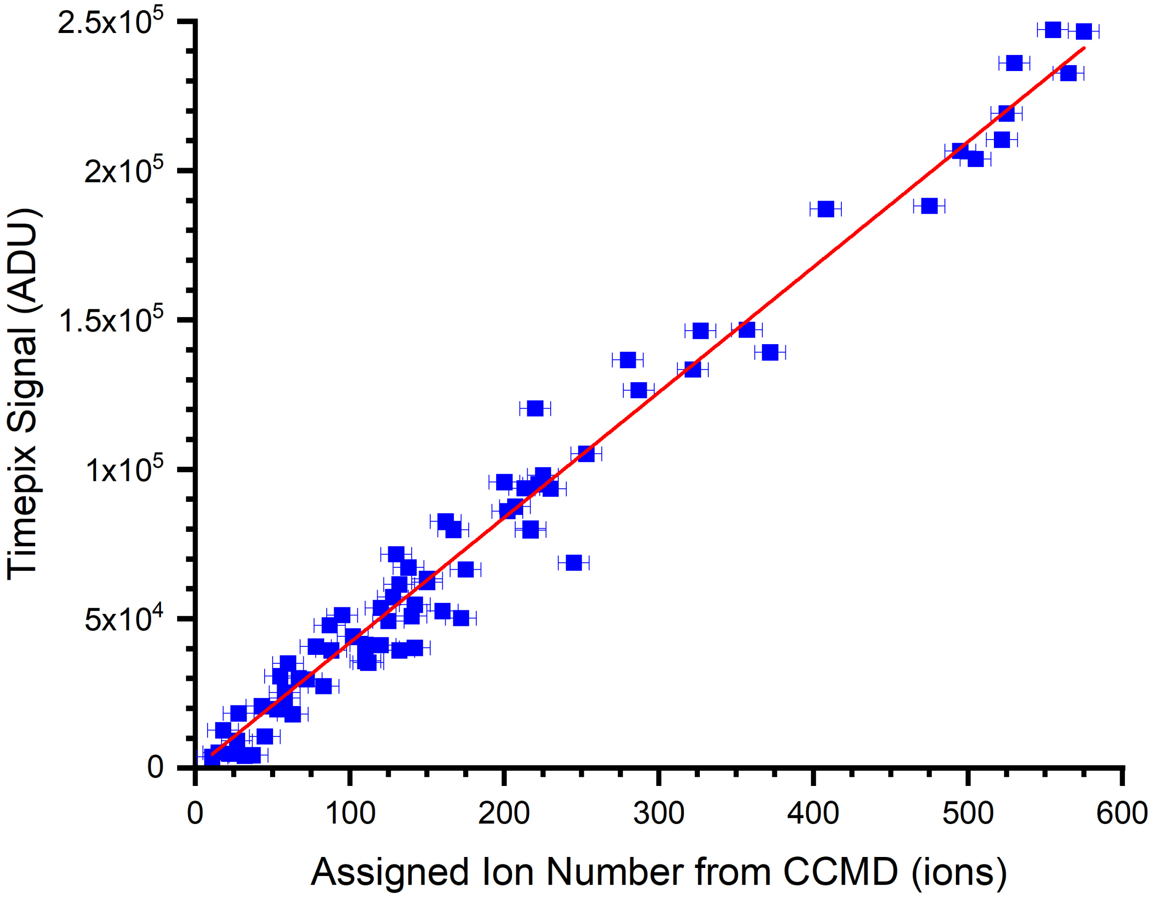

Figure S7: The integrated Ca^+ peak obtained from the Timepix3 mass spectrum is plotted against the ion numbers assigned via comparison of experimental fluorescence images with CCMD simulations, for mono-component crystals. A clear linear relationship can be seen.

ensure the detection efficiency is not overstated.

It is not possible to undertake an equivalent in-depth analysis of multi-component Coulomb crystals in this work, given the relatively short length of the flight tube and the response time of the MCPs. For multi-component crystals, the (unknown) recovery time of the MCPs is important due to its influence on the 'double hit' rate; the response time of the MCPs will have more impact on the detection efficiency of species with higher m/z ratios compared to those with lower m/z ratios. While we anticipate a similar trend to hold, further data is required to quantify the detection efficiencies of the different ionic species, as a function of variable parameters including ion numbers, m/z, V_{rf}, and V_{end}. Undertaking further measurements

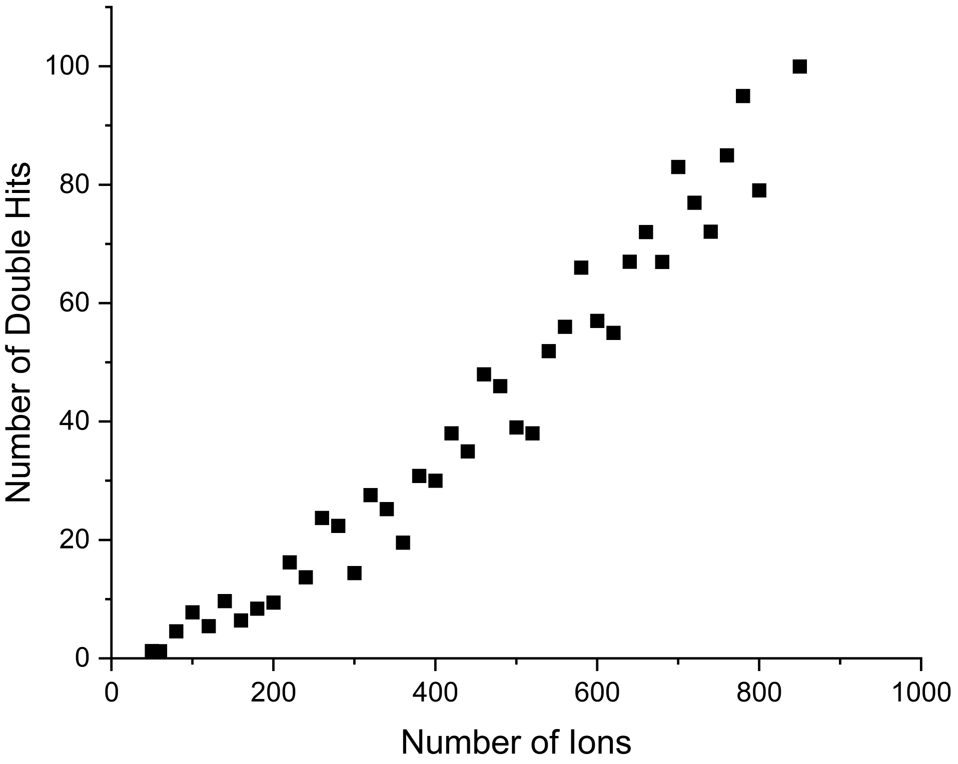

Figure S8: The number of potential double hits on the MCPs (as defined in the text) is plotted against the number of Ca^+ ions in the crystal, based on the calculated SIMION trajectories of CCMD-generated Coulomb crystals.

using an apparatus with a longer flight tube, to increase the temporal separation of different m/z components, and with ion optics in place, to better match the spatial distribution of the ions to the detector area, would be advantageous.

Sensitivity to Ion Positions

As a consequence of experimental imperfections, the centre of the confining electric fields may not be perfectly aligned with the geometric centre of the ion trap. A y-axis offset of approximately 0.4 mm between the two trap centres is identified in this work, established by SIMION simulations and confirmed through experimental variation of the offset fields

applied to the trap rods. Figures S9 and S10 show the simulated spatial distribution of ions at the MCPs when different offsets are applied along the z-axis (a-g) and y-axis (h-l), for a selected Coulomb crystal ($Ca^+ = 130$, $H_3O^+ = 10$, $H_2O^+ = 10$, and $Kr^+ = 12$ ions) using our experimental trapping and ejection fields. The probabilities that the ions reach the detector (as established by SIMION, excluding the effects of the grounded mesh and potential double strike events for clarity) are provided for each of the relative offsets in Table S2.

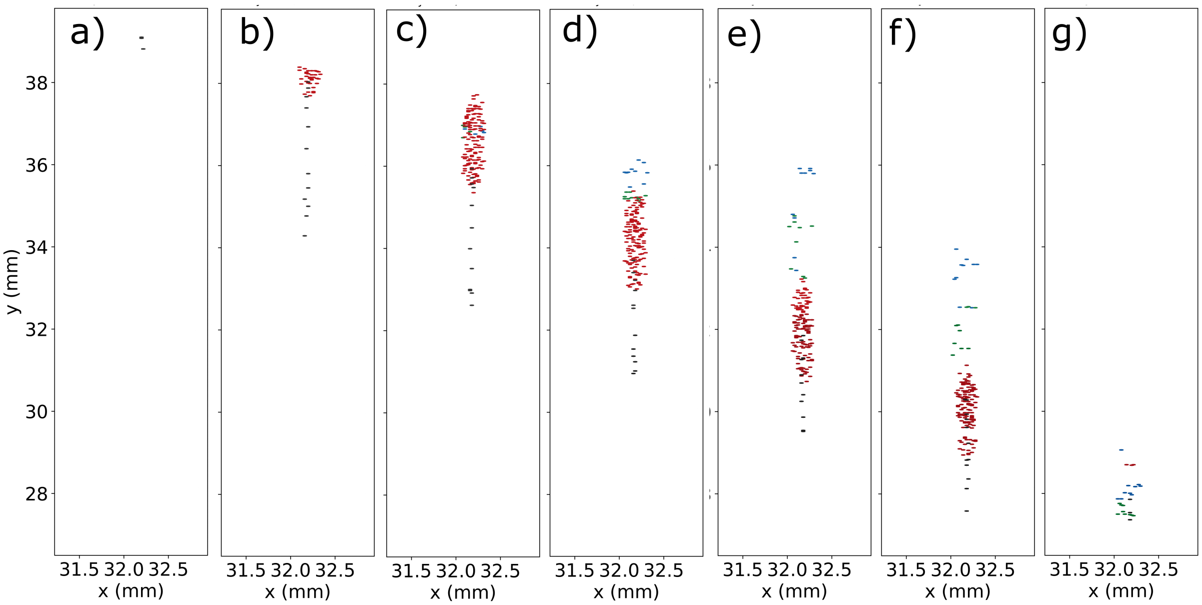

Figure S9: Calculated spatial distributions of ions ejected from the trap with different z-axis offsets away from the geometric trap centre, generated from the SIMION trajectories of the same Coulomb crystal ($Ca^+ = 130$, $H_3O^+ = 10$, $H_2O^+ = 10$, and $Kr^+ = 12$ ions). A fixed y-axis offset of -0.4 mm is applied throughout. Ionic species can be identified by colour: Ca^+ ions are red, H_3O^+ ions are green, H_2O^+ ions are blue, and Kr^+ ions are black.

For a z-axis offset, there is a window from 0 to $+0.25$ mm where 100% of the ions reach the detector; outside this window, ions start hitting the trap rods. (Note that the y-axis offset is set to -0.4 mm when the z-axis offset is varied, as this is the y offset identified in our set-up.) For y-axis offsets between 0 and -0.5 mm, with no offset in the z-axis, all ions reach the detector. When the offset is in the other ($+y$) direction, the detection efficiency of

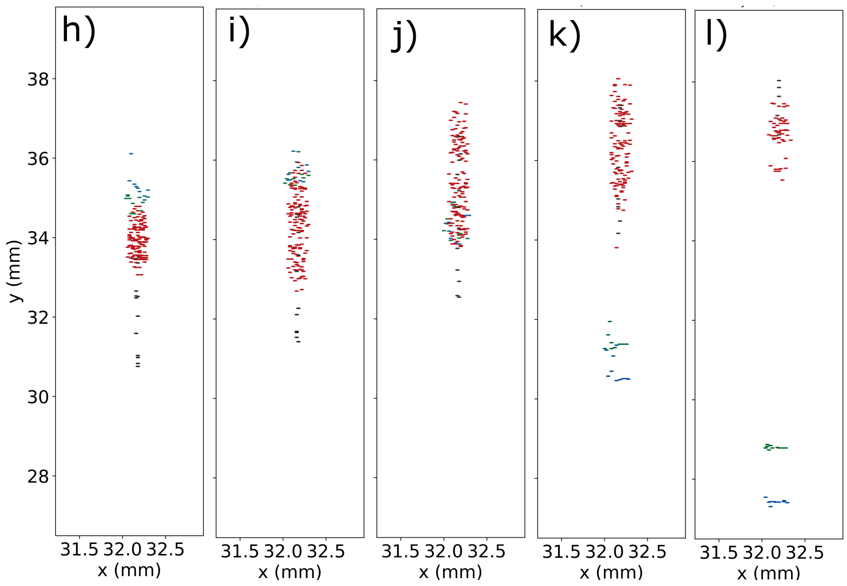

Figure S10: Calculated spatial distributions of ions ejected from the trap with different y-axis offsets away from the geometric trap centre, generated from the SIMION trajectories of the same Coulomb crystal (Ca^+ = 130, H_3O^+ = 10, H_2O^+ = 10, and Kr^+ = 12 ions). A fixed z-axis offset of 0 mm is applied throughout. Ionic species can be identified by colour: Ca^+ ions are red, H_3O^+ ions are green, H_2O^+ ions are blue, and Kr^+ ions are black.

Table S2: The percentages of ions that reach the MCPs in SIMION simulations are provided for a series of y and z offsets, corresponding to the SIMION plots given in Figures S9 and S10. The offset identified in our experimental apparatus is ($y = 0$, $z = -0.4$).

	Offset in z (mm)	Offset in y (mm)	Ions reaching the detector (%)			
			H_2O^+	H_3O^+	Ca^+	Kr^+
(a)	−0.5	−0.4	0%	0%	0%	25%
(b)	−0.25	−0.4	0%	0%	32%	100%
(c)	−0.125	−0.4	100%	50%	100%	100%
(d)	0	−0.4	100%	100%	100%	100%
(e)	0.125	−0.4	100%	100%	100%	100%
(f)	0.25	−0.4	100%	100%	100%	100%
(g)	0.5	−0.4	100%	90%	3%	33%
(h)	0	−0.5	100%	100%	100%	100%
(i)	0	−0.25	100%	100%	100%	100%
(j)	0	0	100%	100%	100%	100%
(k)	0	0.25	100%	100%	88%	75%
(l)	0	0.5	100%	100%	27%	42%

Ca^+ and Kr^+ falls as some ions strike the trap rods or other surfaces and do not reach the MCPs. The lack of symmetry in the positive and negative offsets arises due to the use of

experimental trapping and ejection fields, where there are asymmetries present (as detailed in the main text).

References

[1] T. Poikela, J. Plosila, T. Westerlund, M. Campbell, M. De Gaspari, X. Llopart, V. Gromov, R. Kluit, M. Van Beuzekom, F. Zappon, et al. Timepix3: a 65K channel hybrid pixel readout chip with simultaneous ToA/ToT and sparse readout. *Journal of Instrumentation*, 9(05):C05013, 2014.

[2] Lambert Instruments. Phosphors, phosphor decay time. 2023. URL: `https://www.lambertinstruments.com/phosphors`. Accessed 05/02/2024.
